# Therapeutic potential of *Bifidobacterium breve* strain A1 for preventing cognitive impairment in Alzheimer’s disease

**DOI:** 10.1038/s41598-017-13368-2

**Published:** 2017-10-18

**Authors:** Yodai Kobayashi, Hirosuke Sugahara, Kousuke Shimada, Eri Mitsuyama, Tetsuya Kuhara, Akihito Yasuoka, Takashi Kondo, Keiko Abe, Jin-zhong Xiao

**Affiliations:** 1 Morinaga Milk Industry Co., Ltd Next Generation Science Institute, 5-1-83 Higashihara, Zama, Kanagawa 252-8583 Japan; 2 Group for Food Functionality Assessment, Kanagawa Institute of Industrial Science and Technology, 3-25-13 Tonomachi, Kawasaki-ku, Kawasaki, Kanagawa 210-0821 Japan; 3 Department of Applied Biological Chemistry, Graduate School of Agricultural and Life Sciences, The University of Tokyo, 1-1-1, Yayoi, Bunkyo-ku, Tokyo, 113-8657 Tokyo, Japan

## Abstract

It has previously been shown that the consumption of probiotics may have beneficial effects not only on peripheral tissues but also on the central nervous system and behavior via the microbiota–gut–brain axis, raising the possibility that treatment with probiotics could be an effective therapeutic strategy for managing neurodegenerative disorders. In this study, we investigated the effects of oral administration of *Bifidobacterium breve* strain A1 (*B. breve* A1) on behavior and physiological processes in Alzheimer’s disease (AD) model mice. We found that administration of *B. breve* A1 to AD mice reversed the impairment of alternation behavior in a Y maze test and the reduced latency time in a passive avoidance test, indicating that it prevented cognitive dysfunction. We also demonstrated that non-viable components of the bacterium or its metabolite acetate partially ameliorated the cognitive decline observed in AD mice. Gene profiling analysis revealed that the consumption of *B. breve* A1 suppressed the hippocampal expressions of inflammation and immune-reactive genes that are induced by amyloid-β. Together, these findings suggest that *B. breve* A1 has therapeutic potential for preventing cognitive impairment in AD.

## Introduction

Alzheimer’s disease (AD) is a progressive and irreversible neurodegenerative disease that results in gradual cognitive impairment and eventually leads to dementia. However, despite AD being one of the most prevalent neurodegenerative diseases in aging societies, no clinically successful therapeutic strategies for its treatment or prevention have been reported to date.

AD is characterized pathologically by the accumulation of neurofibrillary tangles of hyperphosphorylated tau and senile plaques that are mainly composed of amyloid-β (Aβ)^1^. Aβ is thought to be associated with oxidative damage and neuroinflammation in the brain, which leads to the loss of neurons and progression of the disease^2,3^. Aβ production and accumulation usually begin in around the age of 40, but may take more than 20 years to manifest as cognitive impairment^4^. Therefore, disease progression is too advanced for treatment once AD has become clinically obvious. This highlights the importance of preventing the onset of AD through improvements in lifestyle or diet^5^.

Probiotics are living microorganisms that are known to confer health benefits onto the host when ingested in adequate amounts^6^. Some probiotic strains appear to have health-promoting effects such as improvement of the intestinal environment, anti-obesity effects^7^, cancer-preventing effects^8^, immunomodulatory functions^9^, prevention of infections^10^, and extension of lifespan^11^. Surprisingly, some probiotics also appear to influence the central nervous system (CNS) and behavior via the microbiota–gut–brain axis^12^. For example, Liu *et al*.^13^ reported that treatment with *Lactobacillus plantarum* PS128 normalized anxiety-like behavior in mice that had been subjected to early life stress and reduced inflammatory cytokine levels in the plasma. Moreover, Distrutti *et al*.^14^ found that treatment with VSL#3, a probiotics mixture containing eight different Gram-positive bacterial species, modulated neuronal functions and long-term potentiation in young and aged rats, and could alter the expression of genes associated with inflammation and neural plasticity in the brain tissue, such as brain-derived neurotrophic factor and synapsin. Moreover, It has been reported that consumption of a mixture of probiotics could affect cognitive function and some metabolic statuses in AD patients^15^. Therefore, it is possible that some probiotics could enable the effective therapeutic management of neurodegenerative disorders.

In the present study, we investigated the effect of *Bifidobacterium breve* strain A1 (*B. breve* A1) on the behaviors and physiological processes of AD model mice. We found that this probiotic can prevent the cognitive dysfunction induced by Aβ, indicating its therapeutic potential in AD patients.

## Results

### *B. breve* A1 prevents Aβ-induced cognitive dysfunction

**Figure 1:**
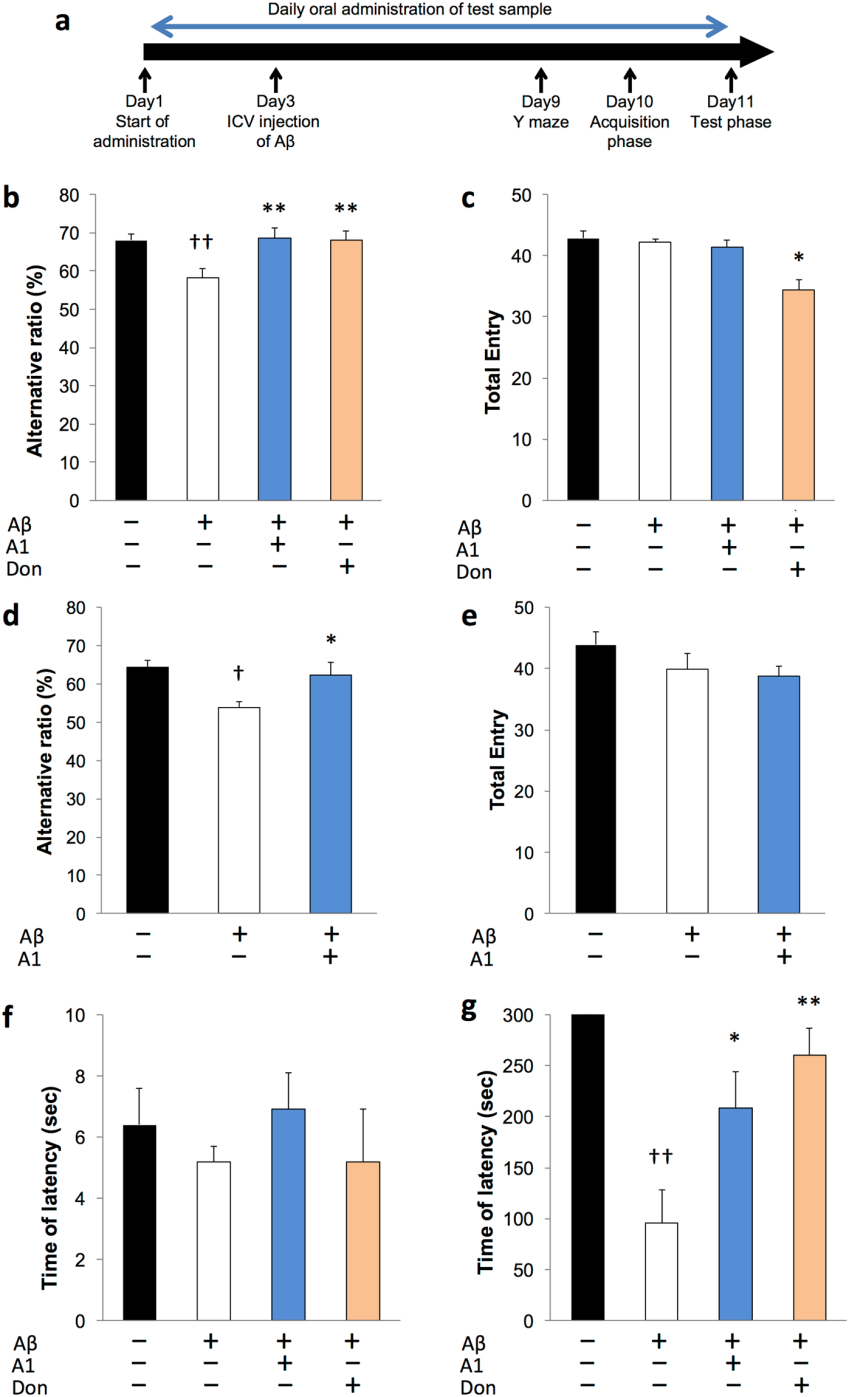
Effect of *Bifidobacterium breve* strain A1 treatment on cognitive function in AD model mice evaluated by Y maze test and passive avoidance test. (**a**) Experimental design of the mouse study. An animal model of AD was induced by intracerebroventricular (ICV) injection of Aβ25–35 or Aβ1–42. The probiotics was orally administered every day starting 2 days before ICV injection. 6 days after ICV, cognitive function was evaluated by Y maze test, thereafter the mice received passive avoidance test. (**b**) Alternative ratio in Y maze test. (**c**) Total entry time in Y maze test. (**d**) Alternative ratio and (**e**) Total entry time in Y maze test of Aβ1–42 injected mice. (**f**) The latency time of acquisition trial. (**g**) The latency time of testing session. For (**b**,**c**,**f**,**g**), mice were injected Aβ25–35. n = 10 mice in each group. For (**d**,**e**), Aβ1–42 was ICV injected, n = 11–12 mice in each group. ^†^P < 0.05, ^††^P < 0.01 vs. control (sham). *P < 0.05, **P < 0.01 vs. Aβ ( + ). All values are expressed as mean ± S.E.. A1: *B. breve* A1, Don: Donepezil.

Figure 1a showed experimental design of this study. Mice that had been intracerebroventricularly administered Aβ25-35 showed a significant reduction of alternation behavior in the Y maze test compared with control mice, indicating that mice with Aβ25–35 showed impaired working memory (Fig. 1b). However, daily administration of *B. breve* A1 or treatment with donepezil, a centrally acting cholinesterase inhibitor, markedly attenuated this alternation behavior impairment to the same level as observed in control mice. There was no significant difference in the total number of entries into the three arms among the groups except for the donepezil group (Fig. 1c), suggesting that *B. breve* A1 did not affect locomotor activity. Similar results were observed in mice that had been intracerebroventricularly administered Aβ1–42 (Fig. 1d,e).

In the passive avoidance test, there was no significant difference in the latency time among groups in the acquisition trial (Fig. 1f). During the test trial, Aβ25–35-injected mice had a significantly lower latency time than control mice, but this was reversed following the daily administration of *B. breve* A1 or donepezil (Fig. 1g), indicating that *B. breve* A1 could ameliorate memory dysfunction in mice administered Aβ.

### *B. breve* A1 suppresses Aβ-induced changes in gene expression in the hippocampus

**Figure 2:**
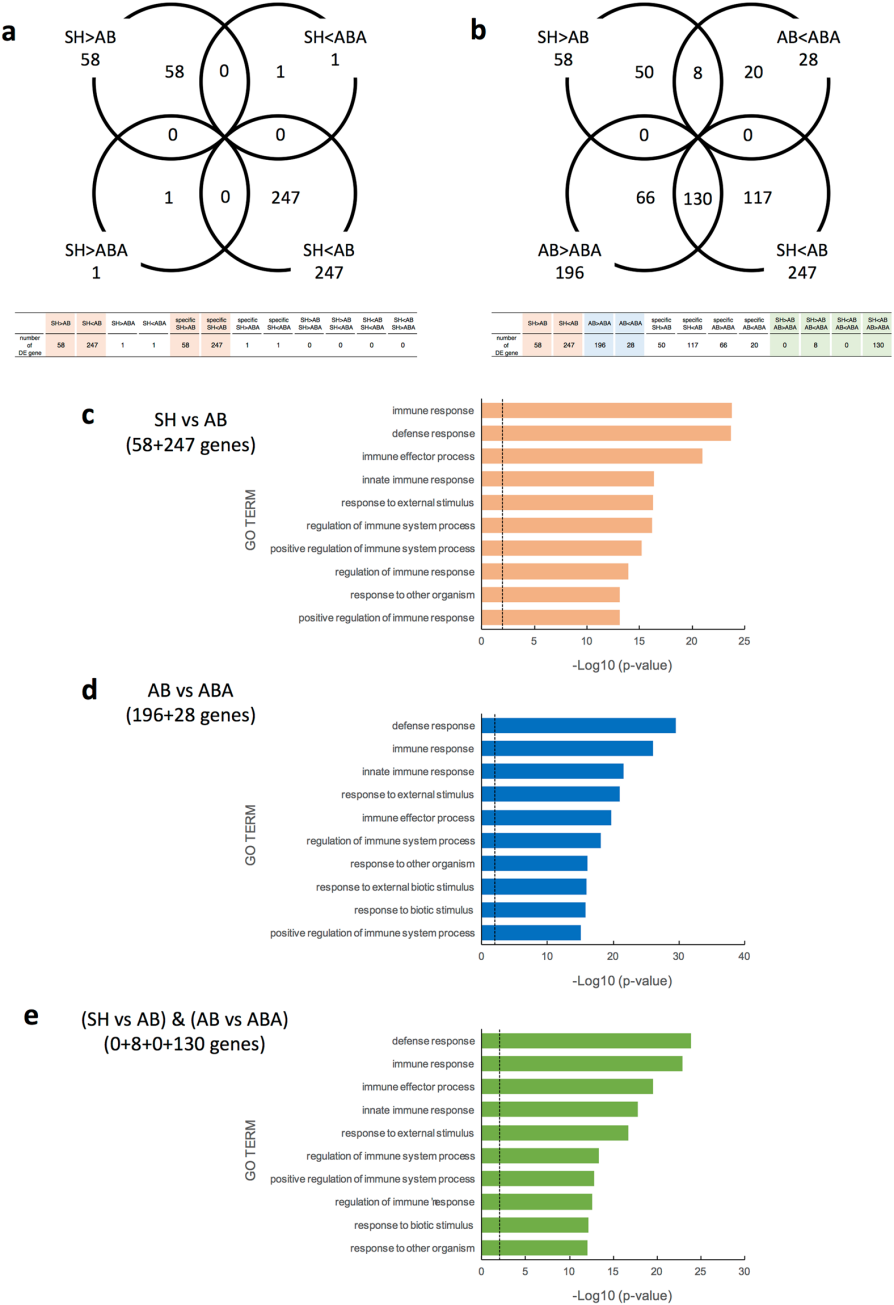
Change of gene expression profile in hippocampus of AD model mice by *Bifidobacterium breve* strain A1 treatment using RNA-seq analysis. Transcriptional analysis was performed on hippocampal tissues of sham-operated mice (SH), Aβ25-35 injected mice (AB) and mice treated with Aβ and *B breve* A1 (ABA). (**a**,**b**) Venn diagram of shared and unique hippocampal transcripts (**a**) in SH vs AB and/or SH vs ABA, and (**b**) in SH vs AB and/or AB vs ABA, p < 0.05 and FDR < 0.05. RNA-seq data from 5 mice are presented. (**c**–**e**) GO Term enrichment analysis of differential expressed (DE) genes in AD hippocampus. Enrichment analysis of differential expressed genes (**c**) between Aβ-treated and control mice, (**d**) between groups with or without *B. breve* A1 administration and (**e**) using DAVID analysis.

To explore gene expression changes in response to administration of *B. breve* A1, transcriptional profiling was performed on hippocampal tissues. In total, 305 genes (247 upregulated, 58 downregulated) were found to be significantly modulated in mice that had been administered Aβ25–35 (AB) compared with sham-operated mice (SH) (Fig. 2a, Supplemental Fig. 1a,b and Supplemental Table 1). DAVID analysis showed that the differential expressed (DE) genes were mainly involved in immune response associated processes, such as “immune response”, “defense response”, and “immune effector process” (Fig. 2c).

Surprisingly, comparison of the gene expression profiles in the hippocampus of mice treated with Aβ and *B. breve* A1 (ABA) and SH mice showed that the expression profiles of only two genes (one upregulated and one downregulated) were significantly different (Fig. 2a, Supplemental Table 1). These data indicated that *B. breve* A1 could modulate excessive immune response induced by Aβ injection, leading to ameliorating effect of Aβ toxicity.

ABA group differed in the expression of 224 genes following the administration of *B. breve* A1 compared to AB group (28 upregulated, 196 downregulated in ABA: Fig. 2b,d, Supplemental Table 1). Among these, 8 of the upregulated genes and 130 of the downregulated genes matched the DE genes observed in AB vs SH comparison (Fig. 2b, Supplemental Table 1). These genes were mainly involved in the defense and immune response (Fig. 2d,e).

We selected some genes which were found to be DE genes by the injection of Aβ and involved in immune-response or synapse plasticity, and performed quantitative RT-PCR on the subset of the genes and verified the RNA-seq data (Supplementary Figure 2). In addition, we performed additional RNA-seq analysis and obtained reproducible result of Fig. 2 using another set of mice.

Furthermore, we investigated whether administration of *B. breve* A1 could affect gene expression of hippocampus in sham-operated mouse. RNA-seq analysis revealed that only one gene was found to be significantly modulated in sham-operated mouse in response to administration of *B. breve* A1 (SHA) (Supplemental Table 2), suggesting that almost all gene expression in hippocampus was not influenced by administration of *B. breve* A1 under normal condition.

### *B. breve* A1 does not affect gut microbiota but significantly increases plasma acetate levels in AD model mice

We next investigated the effect of *B. breve* A1 on intestinal microbiota composition by sequencing bacterial 16 S rRNA gene. The three most dominant bacterial phyla were present in all treatment groups, with Firmicutes being the most prevalent, followed by Bacteroidetes and Proteobacteria (Table 1). Bray-Curtis dissimilarity based principal coordinates analysis (PCoA) and two-way permutation MANOVA analysis showed no intergroup difference in the composition of gut microbiota at phylum levels (Supplemental Fig. 3). However, there were some minor changes by probiotic administration; the proportions of phylum Actinobacteria and family *Bifidobacteriaceae* were significantly higher in ABA than in AB; whereas family *Odoribacteraceae* and *Lachnospiraceae* were slightly lower in ABA (Table 1).

**Table 1:** Microbiota profiles in cecal samples of AD mice.

**Phylum**	Median percent of total reads (interquartile range)					
	SH		AB		ABA	
Actinobacteria	0.14	(0.12–0.17)	0.25	(0.17–0.25)	0.62	(0.57–0.81)*
Bacteroidetes	41.60	(35.44–43.20)	37.52	(31.95–38.27)	30.97	(30.41–33.37)
Firmicutes	54.97	(53.00–60.60)	59.01	(58.39–64.39)	64.69	(62.74–65.48)
Proteobacteria	2.61	(2.31–2.72)	2.50	(2.25–2.56)	2.86	(2.30–3.50)
other	0.98	(0.81–0.99)	1.02	(0.80–1.22)	0.62	(0.49–0.69)
**Family**						
*Alcaligenaceae*	0.09	(0.05–0.21)	0.07	(0.04–0.09)	0.14	(0.06–0.2)
*Bacteroidaceae*	6.19	(5.77–7.84)	3.54	(3.16–4.47)	3.32	(3.3–3.64)
Bacteroidales; f__	1.03	(0.75–1.17)	0.66	(0.63–0.75)	0.78	(0.47–1.1)
Bacteroidales; *f__S24-7*	17.78	(17.01–18.28)	22.09	(17.19–22.92)	18.27	(16.45–19.56)
Bacteroidales; Other	0.04	(0.03–0.04)	0.04	(0.03–0.04)	0.02	(0.02–0.03)
*Bifidobacteriaceae*	0.01	(0.01–0.01)	0.02	(0.01–0.02)	0.52	(0.38–0.62)**
*Clostridiaceae*	0.45	(0.25–0.7)	0.35	(0.34–0.36)	0.25	(0.22–0.55)
Clostridiales; Other	0.04	(0.02–0.09)	0.07	(0.06–0.12)	0.12	(0.1–0.13)
Clostridiales;f__	21.18	(20.49–25.58)	25.10	(22.51–29.43)	35.06	(34.81–37.76)
*Coriobacteriaceae*	0.13	(0.12–0.14)	0.23	(0.14–0.24)	0.20	(0.13–0.21)
*Dehalobacteriaceae*	0.12	(0.07–0.15)	0.10	(0.08–0.11)	0.11	(0.1–0.12)
*Desulfovibrionaceae*	2.32	(2.26–2.62)	2.47	(2.11–2.49)	2.55	(2.25–3.29)
*Erysipelotrichaceae*	1.64	(1.07–2.09)	1.18	(0.92–1.88)	1.07	(0.95–1.22)
*Lachnospiraceae*	9.02	(7.42–9.36)	7.98	(7.8–8.47)	6.69	(5.93–7.47)*
*Lactobacillaceae*	16.42	(14.38–17.73)	17.24	(10.46–18.56)	13.41	(10.87–14.58)
Mollicutes; o__RF39; f__	0.34	(0.3–0.45)	0.37	(0.25–0.46)	0.20	(0.12–0.28)
*Paraprevotellaceae*	2.36	(1.34–4.7)	2.00	(1.4–2.33)	3.27	(1.01–4.05)
*Peptococcaceae*	0.07	(0.05–0.07)	0.07	(0.06–0.07)	0.06	(0.06–0.07)
*Porphyromonadaceae*	0.63	(0.51–0.66)	0.50	(0.28–0.53)	0.49	(0.33–0.52)
*Prevotellaceae*	1.01	(0.72–1.17)	1.52	(0.75–1.86)	0.53	(0.38–0.76)
*Rikenellaceae*	6.87	(5.57–7.76)	5.15	(4.49–6.43)	3.90	(3.85–5.98)
*Ruminococcaceae*	7.51	(6.78–8.34)	8.23	(6.5–8.76)	5.01	(4.75–5.89)
*Verrucomicrobiaceae*	0.19	(0.03–0.21)	0.00	(0–0)	0.00	(0–0)
*[Mogibacteriaceae]*	0.20	(0.15–0.21)	0.20	(0.16–0.22)	0.14	(0.13–0.18)
*[Odoribacteraceae]*	0.91	(0.65–1.03)	1.63	(1.44–1.92)	0.57	(0.45–0.85)**
Other	0.41	(0.35–0.46)	0.51	(0.41–0.51)	0.35	(0.3–0.48)

Microbiotal profiles of sham (SH), Aβ25–35 injected mice (AB) and mice treated with Aβ and *Bifidobacterium breve* strain A1 (ABA) at phylum and family level. n = 5 for each group. Inter-group differences were analyzed using the Mann–Whitney U test. *P < 0.05, **P < 0.01 vs. AB.

**Figure 3:**
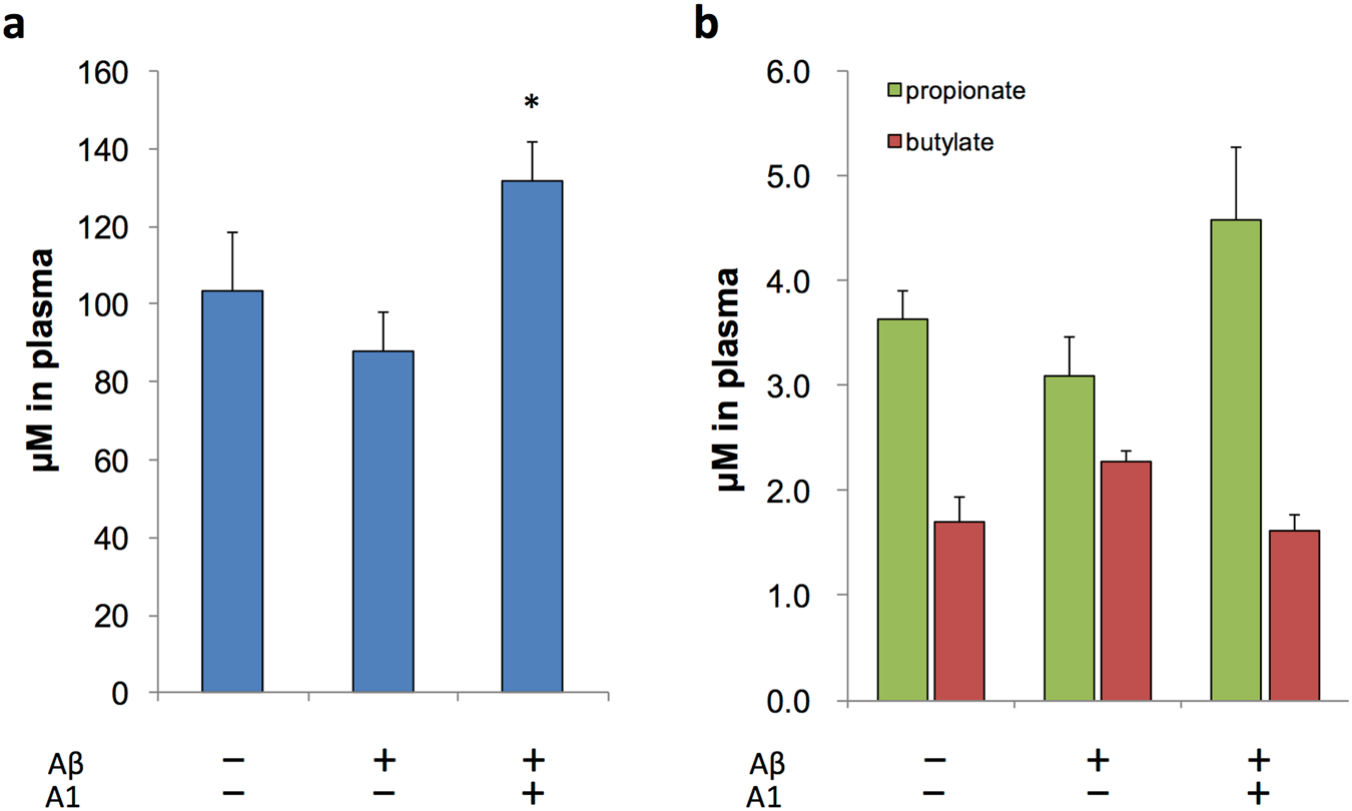
Plasma SCFA levels of AD model mice. (**a**,**b**) Plasma SCFA levels of AD model mice for acetate (**a**) and for propionate and butylate (**b**). N = 4 for each group. *P < 0.05 vs. Aβ ( + ). All values are expressed as mean ± S.E.. A1: *B. breve* A1.

One of the main functions of gut microbiota is the fermentation of dietary fibers in the gut and produce short-chain fatty acids (SCFAs), mainly acetate, propionate, and butyrate, which are the major end products of carbohydrate metabolism in *Bifidobacteria*. Then, we explored SCFAs level in plasma of AD mice treated with or without *B. breve* A1 using gas chromatography-mass spectroscopy (GC-MS). The plasma concentration of acetate, but not propionate or butylate, was significantly higher in ABA group (Fig. 3a,b).

### Non-viable *B. breve* A1 and acetate partially ameliorate behavioral deficits in AD model mice

**Figure 4:**
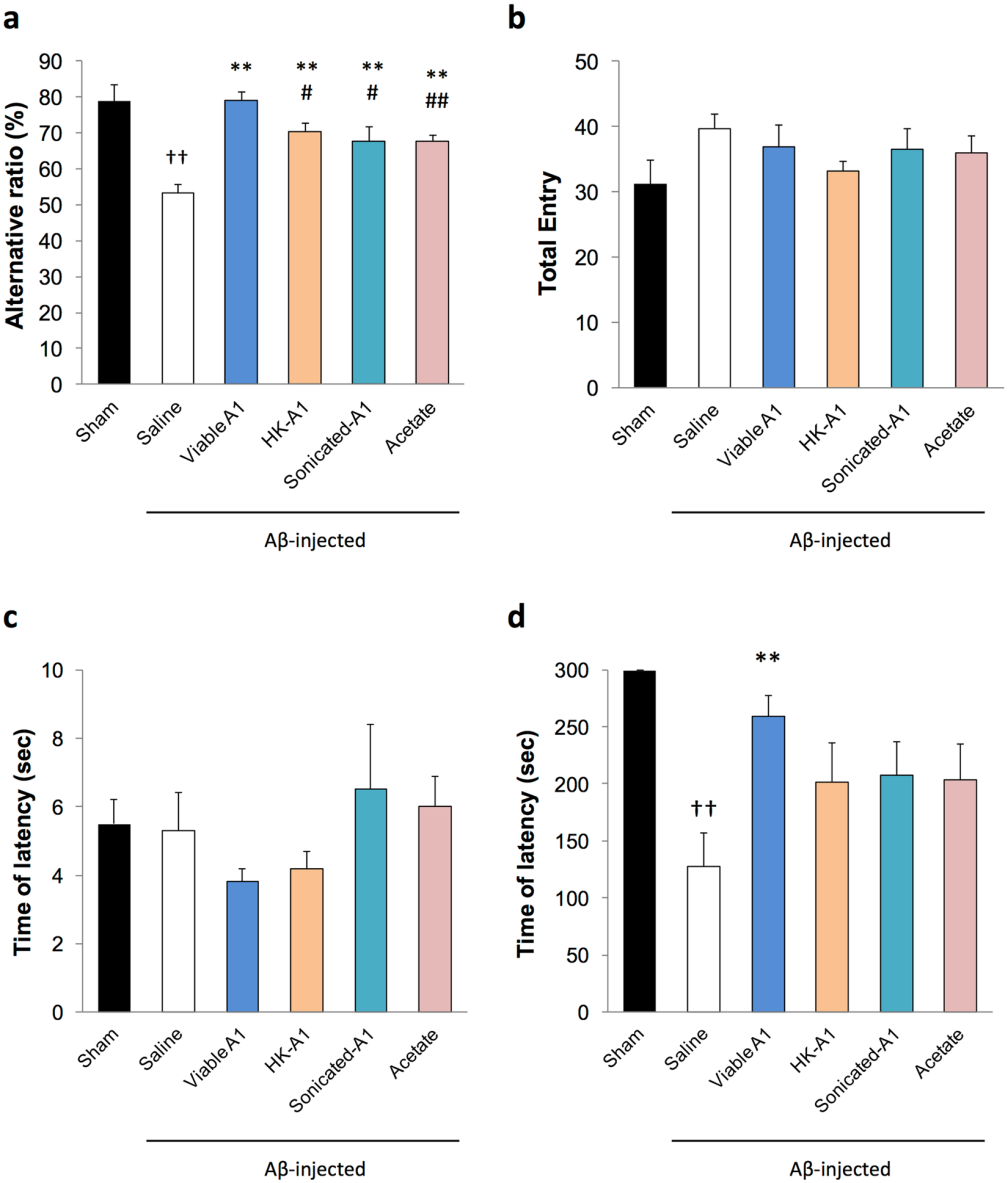
Effect of acetate treatment and non-viable *Bifidobacterium breve* A1 treatment on cognitive function in AD model mice. (**a**) Alternative ratio in Y maze test. (**b**) Total entry time in Y maze test. (**c**) The latency time of acquisition trial. (**d**) The latency time of testing session. Mice were injected Aβ25-35. n = 10 mice in each group. ^††^P < 0.01 vs. control. **P < 0.01 vs. Aβ ( + ). ^#^P < 0.05, ^##^P < 0.01 vs. viable *B. breve* A1 group. All values are expressed as mean ± S.E.

Then, we next explored whether treatment with acetate or non-viable *B. breve* A1 could ameliorate cognitive dysfunction induced by Aβ25–35 using Y maze test and passive avoidance test. In the Y maze test, oral administration with heat-killed or sonicated *B. breve* A1 partially improved the alternation behavior impairment (Fig. 4a). Similarly, the administration of acetate in the drinking water also partially improved the alternation behavior impairment. There was no difference in the total entry time among groups (Fig. 4b). In the passive avoidance test, treatment with heat-killed or sonicated *B. breve* A1, or acetate had no significant effect on the latency time (Fig. 4c,d). Thus, the therapeutic effect of *B. breve* A1 could be partly due to its structural components and the increase in plasma acetate. We also confirmed that plasma acetate level of acetate treated mice was found to be 135.4 ± 11.0 μM (mean ± SE; n = 5), which was similar to the level of *B. breve* A1 treated mice.

## Discussion

Increasing evidence suggests that probiotics can not only ameliorate physiological functions such as the epithelial barrier, gut homeostasis, and the immune response but may also alter brain function, conferring beneficial effects on psychiatric and neurological diseases^13,16^. The underlying mechanism, however, remains unclear. Here we showed that oral administration of *B. breve* A1 prevented cognitive decline in AD model mice, with a reduction in the immune response and neuronal inflammation.

AD is the most common type of dementia and is thought to be caused by the accumulation of Aβ peptides in the brain^1^. Therefore, many researchers have focused on controlling levels of Aβ in the brain and preventing Aβ toxicity. ICV infusion of Aβ in the rodent brain can mimic aspects of AD and can be useful for developing and evaluating potential new drugs for AD^17^; therefore, this model has frequently been used as an AD mouse model. Here, we confirmed that ICV injection of both Aβ25-35 and Aβ1-42 induced cognitive dysfunction, as evaluated by Y maze and passive avoidance tests (Fig. 1). However, interestingly, we found that *B. breve* A1 has ameliorative effects on cognitive dysfunction in both working memory and long-term memory in Aβ-injected mice.

Neuronal inflammation is associated with a broad spectrum of neurodegenerative diseases, including AD^18^. Several studies have shown that Aβ can induce cerebral oxidative stress and activate microglia and astrocytes, leading to neuroinflammation, neuronal injury, and cognitive impairment^19^. Microarray studies using cortical extracts from AD patients have shown that inflammatory/immune response genes are upregulated in AD patients^20,21^, and animal models of AD^22,23^. Similarly, we found that ICV injection of Aβ significantly reshaped gene expression in the hippocampus of mice; genes involved in the immune response and response to external stimuli were upregulated (Fig. 2, Supplemental Table 1). However, surprisingly, we found that nearly all of the DE genes which were observed in Aβ-injected mice were expressed normally in the hippocampus of *B. breve* A1-administered mice (Fig. 2, Supplemental Table 1), indicating that this probiotic suppressed the toxicity induced by Aβ and normalized the gene expression profile. Notably, the expression of *bdnf*, which plays a crucial role in learning and memory processes^24^, was upregulated to normal level by the administration of *B. breve* A1. Therefore, we hypothesize that administration of *B. breve* A1 prevented cognitive decline in AD model mice through its modulating effect on the immune response and neuronal inflammation.

It has previously been suggested that probiotics alter the microbial composition, which could modulate gene expression in the brain^14^. Although proportion of *Bifidobacteriaceae*, *Odoribacteraceae and Lachnospiraceae* changed, we did not detect marked effect of the short-term administration of *B. breve* A1 on overall composition of the intestinal microbiota (Table 1), indicating that some other mechanism must be involved. One possible mechanism could be related to gut–brain communication via stimulation of the vagus nerve, which conveys information from the peripheral organs to the CNS^25^. It has recently been shown that some probiotics modulate anxiety-like behavior through the integrity of the vagus nerve^26,27^ and stimulation of the vagus nerve has been suggested to exert anti-inflammatory effects via the neurotransmitter acetylcholine^28,29^, thus has therapeutic applications for refractory depression, pain and epilepsy^30–32^. Kamiya *et al*.^33^ also reported that administration of viable or non-viable probiotics could prevent the visceral pain that is induced by colorectal distension through their effects on enteric nerves, and that the structural components of probiotics may play an important role in regulating physiological processes. Furthermore, the finding that heat-killed *Lactobacillus brevis* SBC8803 can modulate intestinal vagal nerve activity and autonomic neurotransmission mediated by HT-3 receptors^34^ suggests that non-viable bacterial components may also have potential in modulating neuronal function. Here, we found that non-viable *B. breve* A1 partially recovered cognitive dysfunction in AD mice (Fig. 4), implying that some structural components of the probiotics may modulate the neuronal immune response via vagus nerve stimulation. However, further research is needed to explore whether *B. breve* A1 directly stimulates the vagus nerve, and to clarify the links between probiotics, vagus nerve and immunoregulation.

Metabolite changes that are modulated by the intestinal microbiota could also be involved in the mechanisms. For instance, a metatranscriptome study reported that treatment with some probiotics could affect bacterial metabolic activities in the gut and modulate the host metabolome^35^, which influence CNS function^25,36^. Some metabolites circulating through the body can penetrate the blood–brain barrier, enter the CNS and directly impact neuronal function and regulate peripheral immune function^37^, which could, in turn, modulate CNS function through the brain lymphatic network^38^. SCFAs are the main metabolites of gut microbiota^39^ and have been shown to have immune modulatory functions and decrease colonic inflammation in animal models of inflammatory bowel disease^40,41^. Therefore, it is possible that *B. breve* A1 consumption modulated the immune response in the brain through the production of SCFAs. We found that the administration of *B. breve* A1 increased plasma acetate levels (Fig. 3) and the addition of acetate to drinking water resulted in partial cognitive improvement in AD mice (Fig. 4), indicating that the protective effects of *B. breve* A1 may partly be mediated by the enhanced production of acetate. The mechanism of how acetate ameliorate memory dysfunction in AD mice is one of the important issues to be addressed in future study.

Until now, no clinically successful therapeutic treatment or drug for AD has been reported to date. By the time AD is diagnosed, its progression is too advanced for treatment, making a therapy that reduces Aβ production or suppresses Aβ toxicity through oxidative damage or neuronal inflammation particularly attractive. Although we could not explore whether *B. breve* A1 suppresses Aβ accumulation in the present study, we demonstrated that oral administration of *B. breve* A1 to AD model mice not only improves cognitive function but also suppresses the expression of inflammation and immune-reactive genes that are induced by Aβ, suggesting that *B. breve* A1 has therapeutic potential for preventing cognitive impairment in AD. Furthermore, *B. breve* A1 is likely to be a good candidate for the long-term treatment of AD because *Bifidobacterium* is generally recognized as safe and can be daily ingested for long term. However, since the AD model used in this study does not reflect the usual biological processes of AD such as Aβ burden, further research is required to investigate the effect of *B. breve* A1 on Aβ processing or deposition in other AD models such as traditional APP-overexpression mouse^42^ or recently reported APP knock-in mouse^43^. In addition, additional investigations to clarify the preventive effect of *B. breve* A1 using tauopathy model mouse are also the important issues to be addressed in future studies.

In conclusion, the present study demonstrated that oral administration of *B. breve* A1 to AD model mice not only improved cognitive dysfunction but also suppressed the expression of inflammation and immune-reactive genes induced by Aβ. These results suggest therapeutic potential of *B. breve* A1 for preventing cognitive impairment in AD.

## Methods

All procedures were performed in accordance with the National Institutes of Health guidelines for the use of experimental animals. The experimental protocol was reviewed and approved by the Animal Studies Committee of Nihon Bioresearch Inc. and the Animal Research Committee of Morinaga Milk Industry Co., Ltd, Japan.

### Preparation of probiotics

*B. breve* A1 was isolated from the feces of human infants, and identified by its cellular morphology, sugar fermentation pattern, and 16 S rRNA sequence. *B. breve* A1 was cultured in a medium containing glucose, yeast extract and salts. The cells were harvested by centrifugation, washed and lyophilized. Lyophilized *B. breve* A1 was suspended in saline at a concentration of 5 × 10^9^ cfu/ml for experimental use. Heat-killed *B. breve* A1 was prepared by heat-shocking the bacterium at 60 °C for 60 min and was stored at −20 °C until use. Sonicated *B. breve* A1 was prepared by suspending the bacterium in saline, sonicating (output control 4, constant) for 60 min on ice using SONIFIER® 450 (BRANSON, Danbury, Tewksbury, Conn., U.S.A.) and centrifuging the homogenate at 800 × g for 30 min at 4 °C; the supernatant was then collected and stored at −20 °C until use. We confirmed that the heat-treated and sonicated samples contained no living *B. breve* A1 by plate counting.

### Animals and Treatments

Male 10-week-old ddY mice (SLC, Inc., Shizuoka, Japan) were housed in a room with controlled lighting (12 h light/12 h dark) and a constant temperature (25 °C), and provided with MF diet (Oriental Yeast Co., Ltd., Tokyo, Japan) and water *ad libitum*.

Living, heat-killed or sonicated *B. breve* A1 were orally administered to the mice daily by gavaging 1 × 10^9^ organisms in 0.2 ml, starting 2 days before Aβ injection. For acetate group, mice were administered sodium acetate (150 mM) in drinking water from 2 days before Aβ injection (acetate group). As a positive control group, mice were orally administered donepezil hydrochloride (0.5 mg^−1^ kg^−1^ day^−1^; Wako Chemicals, Osaka, Japan). No adverse effects were observed following administration of any of the sample solutions.

Aβ protein 25–35 (Peptide Institute, Osaka, Japan) was dissolved in distilled water (final concentration 2 mM) and incubated at 37 °C for 96 h. ICV injection was performed as described previously^44^ with some modification. Briefly, each mouse was anesthetized by intraperitoneal injection of Nembutal in saline and subcutaneous injection of levobupivacaine, and placed in a stereotaxic frame (Narishige Inc., Tokyo, Japan). 28-gauge needle was inserted to following position: 1mm right of the midline, 0.2 mm posterior and 2.5 mm depth from bregma. Aβ25–35 solution (3 μl, 6 nmol) was then injected intracerebroventricularly at a rate of 1 μl/min using a syringe pump. The needle was kept in place for additional 3 minutes and then withdrawn.

Aβ protein 1–42 (Peptide Institute, Osaka, Japan) was injected into other mice at 200 pmol in 3 μl distilled water, while 3 μl distilled water was injected into a sham-operated group. Aβ 1–42 solution used in this study contained mixture of monomeric and oligomer form of Aβ (Supplemental Fig. 4).

### Behavioral tests

A Y maze test was performed 6 days after ICV injection to assess the working memory of the mice. The maze consisted of polyvinyl plastic and had three arms (395 mm deep, 120 mm high, 45 mm wide at the bottom, 100 mm wide at the top) at angles of 120°. Mice were placed at the end of one arm and allowed to move freely for 7 min. The sequence of arm entry was counted manually to calculate the total number of entries and the alternation ratio (ratio of actual alternations to maximum alternations, i.e., total number of entries −2). This test was performed by a person blind to the group assignment.

One day after the Y maze test, the long-term memory of mice was evaluated by a passive avoidance test. The apparatus consisted of one illuminated (100 mm wide, 100 mm deep, 300 mm high) and one dark (240 mm wide, 245 mm deep, 300 mm high) chamber with grid floors, which were separated by a guillotine door. During the acquisition trial, each mouse was placed in the illuminated chamber, the guillotine door was opened after 10 s and the initial latency to enter the dark compartment was recorded. When the mice had moved completely into the dark compartment, the door was closed and the mice received an electric shock (0.2 mA, 2 s duration, scrambled). They were then returned to their home cage. The test trial was conducted 24 h later by placing the mice in the illuminated chamber and measuring the latency period to enter the dark compartment up to 300 s.

### Physiological analyses

Following testing, mice were euthanized by isoflurane overdose, and the hippocampus was removed, frozen in liquid nitrogen and stored at −80 °C until use. Blood was collected into a tube containing EDTA and centrifuged at 2150 × g at 4 °C for 15 min. Subsequently the plasma sample was frozen in liquid nitrogen and stored at −80 °C until analysis. The cecum was also removed and its contents were stored at −80 °C until use.

### RNA sequencing (RNA-seq) analysis

Total RNA was extracted from the hippocampi with the RNeasy® Plus Universal Mini Kit (Qiagen,Venlo, Netherlands) according to the manufacturer’s instructions. Poly(A)-selected RNA-seq libraries were generated using the TruSeq RNA Sample Prep Kit V2 (Illumina, San Diego, CA) and 150-bp paired-end sequencing was performed at BGI JAPAN Co. Ltd. using the HiSeq System (Illumina). Reads with adaptors, >5% unknown bases and low quality (i.e., >20% bases with quality <15) were filtered.

The reads from each sample were aligned to the *Mus musculus* genome GRCm 38.73 assembly using the RNA-seq pipeline from CLC bio (CLC bio, Tokyo, Japan). Unique read counts for annotated mouse genes in the database were then analyzed using the R package DESeq. 2 (version 3.3.2)^45^. Differentially expressed (DE) genes were identified at a combined cut-off of *P* < 0.05 and a false discovery rate (FDR) < 0.05. The Database for Annotation, Visualization and Integrated Discovery (DAVID) was used to add functional annotation to the DE gene lists and to statistically assess the annotations^46^.

### Microbiota analysis

DNA was extracted from the cecal samples using the bead-beating method and 16 S rRNA gene sequencing was performed as described previously with slight modifications^47^. The detailed description of the methods is provided in Supplementary Information.

### SCFA analysis

The plasma samples were prepared for GC-MS analysis as described previously^48^ with modifications. The detailed description of the methods is provided in Supplementary Information.

### Statistical analysis

The behavior and physiological responses of treatment groups were compared using one-way analysis of variance followed by Student’s t or Mann–Whitney U post hoc tests in PASW Statistics for Windows version 17 (SPSS Japan). Statistical analysis for RNA-seq was described in section “RNA sequencing (RNA-seq) analysis”.

### Data availability

The datasets generated and analyzed during the current study are available from the corresponding author on reasonable request.

## Electronic supplementary material

Supplemental Information

## References

[1] Glenner GG, Wong CW (1984). Alzheimer’s disease: initial report of the purification and characterization of a novel cerebrovascular amyloid protein. Biochem. Biophys. Res. Commun..

[2] McNaull BBA, Todd S, McGuinness B, Passmore AP (2010). Inflammation and anti-inflammatory strategies for Alzheimer’s Disease – A mini-review. Gerontology.

[3] Wyss-Coray T (2006). Inflammation in Alzheimer disease: driving force, bystander or beneficial response?. Nat. med.

[4] Jack CR (2010). Hypothetical model of dynamic biomarkers of the Alzheimer’s pathological cascade. Lancet Neurol..

[5] Pistollato F (2016). Role of gut microbiota and nutrients in amyloid formation and pathogenesis of Alzheimer disease. Nutr. Rev..

[6] Gareau MG, Sherman PM, Walker WA (2010). Probiotics and the gut microbiota in intestinal health and disease. Nat. Rev. Gastroenterol. Hepatol..

[7] Kondo S (2010). Antiobesity effects of *Bifidobacterium breve* strain B-3 supplementation in a mouse model with high-fat diet-induced obesity. Biosci. Biotechnol. Biochem..

[8] Sivan A (2015). Commensal *Bifidobacterium* promotes antitumor immunity and facilitates anti-PD-L1 efficacy. Science.

[9] Savilahti E (2011). Probiotics in the Treatment and Prevention of Allergies in Children. Biosci. Microflora.

[10] Kafshdooz T (2016). Role of Probiotics in Managing of Helicobacter Pylori Infection: A Review. Drug Res. (Stuttg)..

[11] Matsumoto M, Kurihara S, Kibe R, Ashida H, Benno Y (2011). Longevity in mice is promoted by probiotic-induced suppression of colonic senescence dependent on upregulation of gut bacterial polyamine production. PLoS One.

[12] Sampson TR, Mazmanian SK (2015). Review control of brain development,function,and behavior by the microbiome. Cell Host Microbe.

[13] Liu YW (2016). Psychotropic effects of *Lactobacillus plantarum* PS128 in early life-stressed and naïve adult mice. Brain Res..

[14] Distrutti E (2014). Modulation of intestinal microbiota by the probiotic VSL#3 resets brain gene expression and ameliorates the age-related deficit in LTP. PLoS One.

[15] Akbari E (2016). Effect of probiotic supplementation on cognitive function and metabolic status in Alzheimer’s Disease: a randomized, double-blind and controlled trial. Front. Aging Neurosci..

[16] Ait-Belgnaoui A (2014). Probiotic gut effect prevents the chronic psychological stress-induced brain activity abnormality in mice. Neurogastroenterol. Motil..

[17] Takeda S (2009). Validation of Aβ1–40 administration into mouse cerebroventricles as an animal model for Alzheimer disease. Brain Res..

[18] Kempuraj D (2016). Neuroinflammation induces neurodegeneration. J. Neurol. Neurosurg. spine.

[19] Lee YJ, Han SB, Nam S-Y, Oh K-W, Hong JT (2010). Inflammation and Alzheimer’s disease. Arch. Pharm. Res..

[20] Tan MG (2009). Genome wide profiling of altered gene expression in the neocortex of Alzheimer’s disease. J. Neurosci. Res..

[21] Wang S, Qaisar U, Yin X, Grammas P (2012). Gene expression profiling in Alzheimer’s disease brain microvessels. J. Alzheimers. Dis..

[22] Arisi I (2011). Gene expression biomarkers in the brain of a mouse model for Alzheimer’s disease: mining of microarray data by logic classification and feature selection. J. Alzheimers. Dis..

[23] Wirz KTS (2013). Cortical beta amyloid protein triggers an immune response, but no synaptic changes in the APPswe/PS1dE9 Alzheimer’s disease mouse model. Neurobiol. Aging.

[24] Leal, G., Bramham, C. R. & Duarte, C. B. In*Vitamins and hormones* **104**, 153–195 (2017). 10.1016/bs.vh.2016.10.004 28215294

[25] Fung TC, Olson CA, Hsiao EY (2017). Interactions between the microbiota, immune and nervous systems in health and disease. Nat. Neurosci..

[26] Bravo JA (2011). Ingestion of *Lactobacillus* strain regulates emotional behavior and central GABA receptor expression in a mouse via the vagus nerve. Proc. Natl. Acad. Sci..

[27] Bercik P (2011). The anxiolytic effect of *Bifidobacterium longum* NCC3001 involves vagal pathways for gut-brain communication. Neurogastroenterol. Motil..

[28] Tracey KJ (2000). Vagus nerve stimulation attenuates the systemic inflammatory response to endotoxin. Nature.

[29] Koopman FA (2016). Vagus nerve stimulation inhibits cytokine production and attenuates disease severity in rheumatoid arthritis. Proc. Natl. Acad. Sci..

[30] Kirchner A, Birklein F, Stefan H, Handwerker HO (2000). Left vagus nerve stimulation suppresses experimentally induced pain. Neurology.

[31] Morris GL, Mueller WM (1999). Long-term treatment with vagus nerve stimulation in patients with refractory epilepsy. The Vagus Nerve Stimulation Study Group E01–E05. Neurology.

[32] Rush AJ (2005). Vagus nerve stimulation for treatment-resistant depression: a randomized, controlled acute phase trial. Biol. Psychiatry.

[33] Kamiya T (2006). Inhibitory effects of *Lactobacillus reuteri* on visceral pain induced by colorectal distension in Sprague-Dawley rats. Gut.

[34] Horii Y (2013). Effects of intraduodenal injection of *Lactobacillus brevis* SBC8803 on autonomic neurotransmission and appetite in rodents. Neurosci. Lett..

[35] Holmes E, Li JV, Marchesi JR, Nicholson JK (2012). Gut microbiota composition and activity in relation to host metabolic phenotype and disease risk. Cell Metab..

[36] Rothhammer V (2016). Type I interferons and microbial metabolites of tryptophan modulate astrocyte activity and central nervous system inflammation via the aryl hydrocarbon receptor. Nat. Med..

[37] Rooks MG, Garrett WS (2016). Gut microbiota, metabolites and host immunity. Nat. Rev. Immunol..

[38] Louveau A (2015). Structural and functional features of central nervous system lymphatic vessels. Nature.

[39] Morrison DJ, Preston T (2016). Formation of short chain fatty acids by the gut microbiota and their impact on human metabolism. Gut Microbes.

[40] Smith PM (2013). The microbial metabolites, short-chain fatty acids, regulate colonic Treg cell homeostasis. Science.

[41] Kumar M, Kissoon-Singh V, Coria AL, Moreau F, Chadee K (2017). Probiotic mixture VSL#3 reduces colonic inflammation and improves intestinal barrier function in Muc2 mucin-deficient mice. Am. J. Physiol. Gastrointest. Liver Physiol..

[42] Schaeffer EL, Figueiro M, Gattaz WF (2011). Insights into Alzheimer disease pathogenesis from studies in transgenic animal models. Clinics.

[43] Saito T (2014). Single App knock-in mouse models of Alzheimer’s disease. Nat. Neurosci..

[44] Min LJ (2017). Administration of bovine casein-derived peptide prevents cognitive decline in Alzheimer disease model mice. PLoS One.

[45] Love MI, Huber W, Anders S (2014). Moderated estimation of fold change and dispersion for RNA-seq data with DESeq. 2. Genome Biol..

[46] Dennis G (2003). DAVID: Database for Annotation, Visualization, and Integrated Discovery. Genome Biol..

[47] Odamaki T (2016). Age-related changes in gut microbiota composition from newborn to centenarian: a cross-sectional study. BMC Microbiol..

[48] Tsukahara T (2014). High-sensitivity detection of short-chain fatty acids in porcine ileal, cecal, portal and abdominal blood by gas chromatography-mass spectrometry. Anim. Sci. J..

