# Supplementary material for: Therapeutic potential of Bifidobacterium breve strain A1 for preventing cognitive impairment in Alzheimer’s disease
Source: Sci Rep. 2017 Oct 18;7:13510. doi: 10.1038/s41598-017-13368-2 (PMC5647431; doi:10.1038/s41598-017-13368-2)
Supplement: Supplementary file 1 — Supplemental Information [file 41598_2017_13368_MOESM1_ESM.pdf]

Supplemental information

Therapeutic potential of Bifidobacterium breve strain A1 for preventing cognitive impairment in Alzheimer's disease

Yodai Kobayashi, Hirosuke Sugahara, Kousuke Shimada, Eri Mitsuyama, Tetsuya Kuhara, Akihito Yasuoka, Takashi Kondo, Keiko Abe and Jin-zhong Xiao

Supplemental Material & Method

Microbiota analysis.

DNA was extracted from cecal samples using the bead-beating method20 and 16S rRNA gene sequencing was performed as described previously[1]. Briefly, The V3–V4 region of bacterial 16S rRNA genes were amplified via PCR with a TaKaRa Ex Taq HS kit (TaKaRa Bio, shiga, Japan) and a primer set of Tru357F (5'-CGCTCTTCCGATCTCTG TACGGRAGGCAGCAG-3') and Tru806R (5'-CGCTCTTCCGATCTGAC GGACTACHVGGGTWTCTAAT-3'). The DNA was amplified according to following program: 94 ^{o}C for 30sec; 30 cycles of 94 ^{o}C for 30 sec, 50 ^{o}C for 30 sec and 72 ^{o}C for 5 min. PCR products was amplified with a 2nd primer set, which is adapted for the Illumina MiSeq (Illumina), by following protocol: 94 ^{o}C for 30sec; 8 cycles of 94 ^{o}C for 30 sec, 50 ^{o}C for 30 sec and 72 ^{o}C for 5 min. Thereafter, equal amounts of amplicons from different samples were pooled

and removed primer-dimer by gel-extraction with the QIAquick PCR Purification Kit. The pooled libraries were sequenced by Illumina MiSeq with a MiSeq v3 Reagent kit (Illumina) in Morinaga Milk Company Co. Ltd.

The obtained sequences were filtered by the bowtie -2 program (ver 2-2.2.4) to remove the reads mapped on PhiX174 sequence. Thereafter, the 3' region of each read with a PHRED quality score of less than 17 was trimmed. Trimmed reads less than 150 bp in length with an average quality score of less than 25 or those lacking paired reads were also removed. The trimmed paired-end reads were combined by the fastq-join script in EA-Utils (ver. 1.1.2–537). Potential chimeric sequences were removed by reference-based chimera checking in USEARCH (ver. 5.2.32) and the gold database (http://drive5.com/otupipe/gold.tz). The non-chimeric sequences were analyzed in the QIIME software package version 1.8.0. The sequences were assigned to operational taxonomic units (OTUs) by open-reference OTU picking with a 97% pairwise identity threshold and the Greengenes reference database (ftp://greengenes.microbio.me/greengenes_release/gg_13_5/gg_13_8_otus.tar.gz).

Short-chain fatty acid (SCFA) analysis.

The plasma samples were prepared for gas chromatography-mass spectroscopy (GC-MS) analysis as described previously[2] with modifications. 90 μL of plasma was mixed with 2 μL of 2.9 mM [2H3] acetate (Cambridge Isotope Laboratories,

Tewksbury, MA, U.S.A.) as an internal standard, deproteinized with 2 μL of 1 mg/μL 5-sulfosalicylic acid and centrifuged at 2000 × g for 10 min. The supernatant was then collected and mixed with 2 μL of 35% HCl followed by 600 μL of diethyl ether. The solution was vortexed for 30 min and then centrifuged for 10 min at 1200 × g at room temperature. The ether layer was collected and 1.6 μL of 1-(tert-butyldimethylsilyl) imidazole (Tokyo Chemical Industry, Tokyo, Japan) was added. The solution was heated at 60 °C for 30 min, and then cooled and transferred to a glass vial for GC-MS analyses. Analysis was conducted with the GC-MS-QP2010 Ultra (Shimadzu, Kyoto, Japan) using electron ionization mode and a CP-Sil 8CB-MS column (30 m × 0.25 mm internal diameter × 0.25 μm; J&W Scientific, Folsom, CA, U.S.A.) for GC separation under the following thermal conditions: 40 °C for 1 min, 70 °C/min until 60 °C, 10 °C/min until 110 °C, and 70 °C/min until 250 °C for 1 min. The mass spectrometer was set in selected ion monitoring mode at m/z 117 for acetate, m/z 120 for [2H3] acetate, m/z 131 for propionate, and m/z 125 for butylate.

Quantitative RT-PCR

Real-time quantitative RT-PCR was performed using PrimeScript RT reagent Kit (Takara Bio, shiga, Japan) and SYBR Premix Ex Taq II (Takara Bio). The level of target gene expression was normalized against expression of a housekeeping gene,

glyceraldehyde-3-phosphate dehydrogenase (GADPH), in each sample. PCR primers were shown in supplemental Table 3.

Aβ 1-42 analysis

Photo-Induced Cross-Linking of Unmodified Proteins (PICUP) and SDS-PAGE were performed as described previously[3]. Aβ separated on SDS-PAGE gels were silver-stained using Silver Quest Silver Staining kit (Invitrogen) according to manufacture instruction.

Reference for Supplemental Information

1. Odamaki, T. *et al.* Age-related changes in gut microbiota composition from newborn to centenarian: a cross-sectional study. *BMC Microbiol.* **16,** 90 (2016).
2. Tsukahara, T. *et al.* High-sensitivity detection of short-chain fatty acids in porcine ileal, cecal, portal and abdominal blood by gas chromatography-mass spectrometry. *Anim. Sci. J.* **85,** 494–498 (2014).
3. Rahimi, F., Maiti, P. & Bitan, G. Photo-Induced Cross-Linking of Unmodified Proteins (PICUP) Applied to Amyloidogenic Peptides. *J. Vis. Exp.* (2009).

doi:10.3791/1071

Supplemental Fig 1

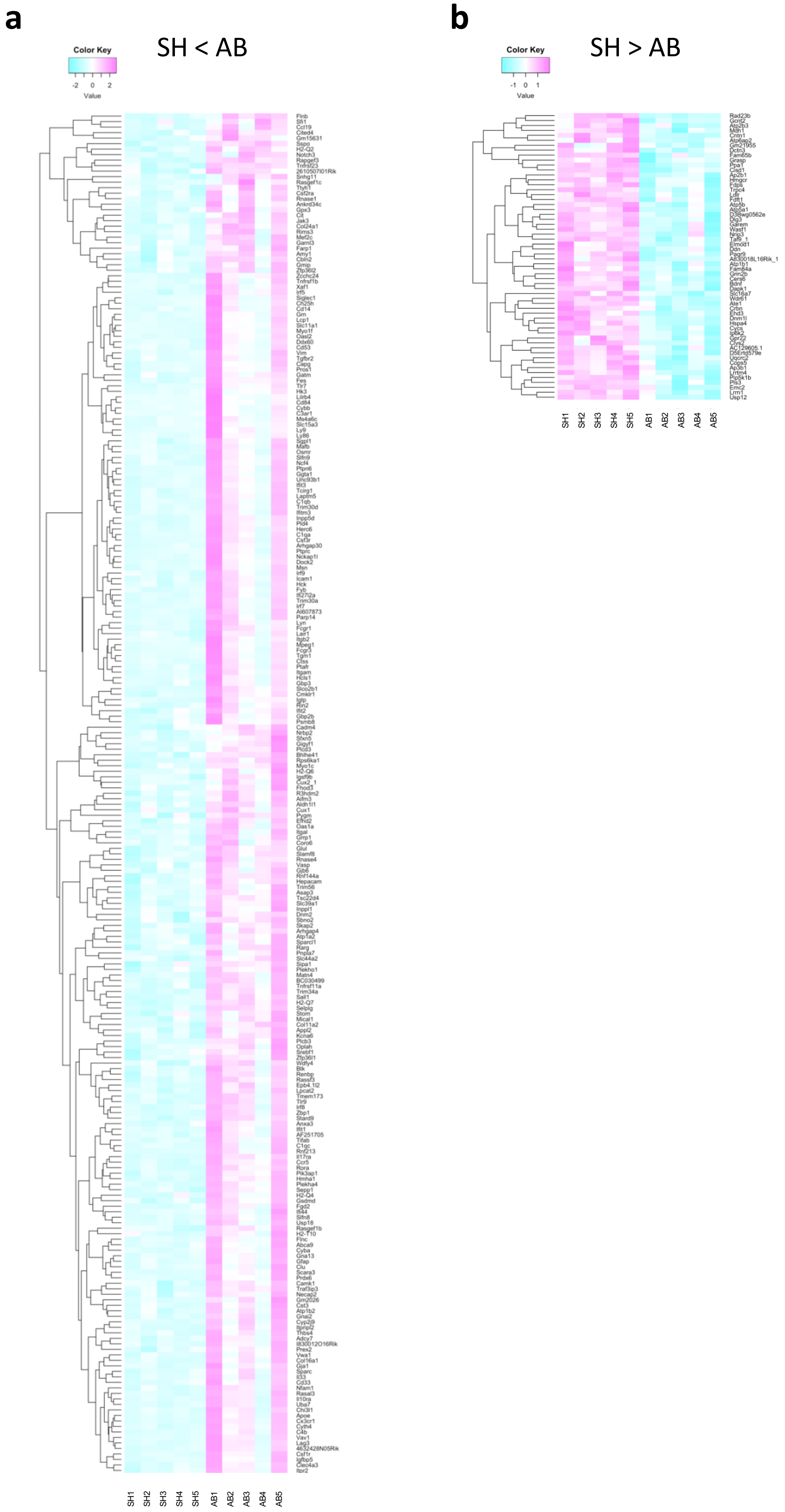

c

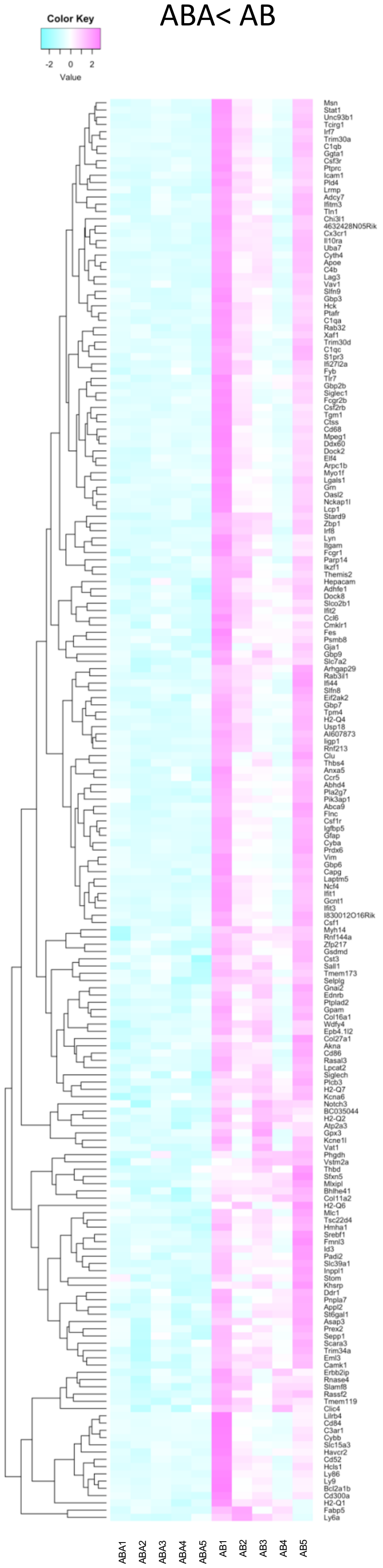

ABA< AB
Color Key
-2 0 2
Value
ABA1
ABA2
ABA3
ABA4
ABA5
AB1
AB2
AB3
AB4
AB5

d

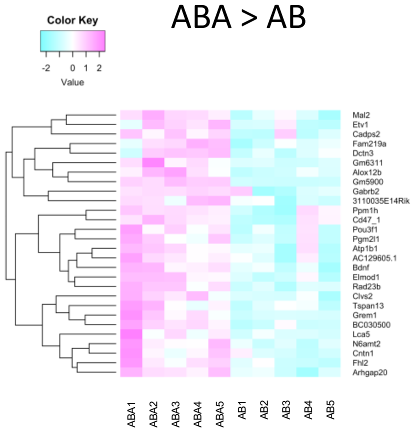

ABA > AB
Color Key
-2 0 1 2
Value
Mal2
Etv1
Cadps2
Fam219a
Dctn3
Gm6311
Alox12b
Gm5900
Gabrb2
3110035E14Rik
Ppm1h
Cd47_1
Pou3f1
Pgm2l1
Atp1b1
AC129605.1
Bdnf
Elmod1
Rad23b
Clvs2
Tspan13
Grem1
BC030500
Lca5
N6amt2
Cntn1
Fhl2
Arhgap20
ABA1
ABA2
ABA3
ABA4
ABA5
AB1
AB2
AB3
AB4
AB5

Supplemental Figure 1.

(a–b) Heatmaps of the significantly modulated gene expression ($p<0.05$ and FDR<0.05 up- or downregulation) in hippocampus of Aβ injected mice (lanes AB1–AB5) in comparison with sham-operated mice (lanes SH1-SH5). We detected changes in the expression of 305 genes (247 up-regulated, 58 down-regulated). (c–d) Heatmaps of the significantly modulated gene expression in hippocampus of Aβ injected mice administrated B. *breve* A1 (lanes ABA1–ABA5) compared with non-administrated mice (lanes AB1–AB5).

Supplemental Fig 2

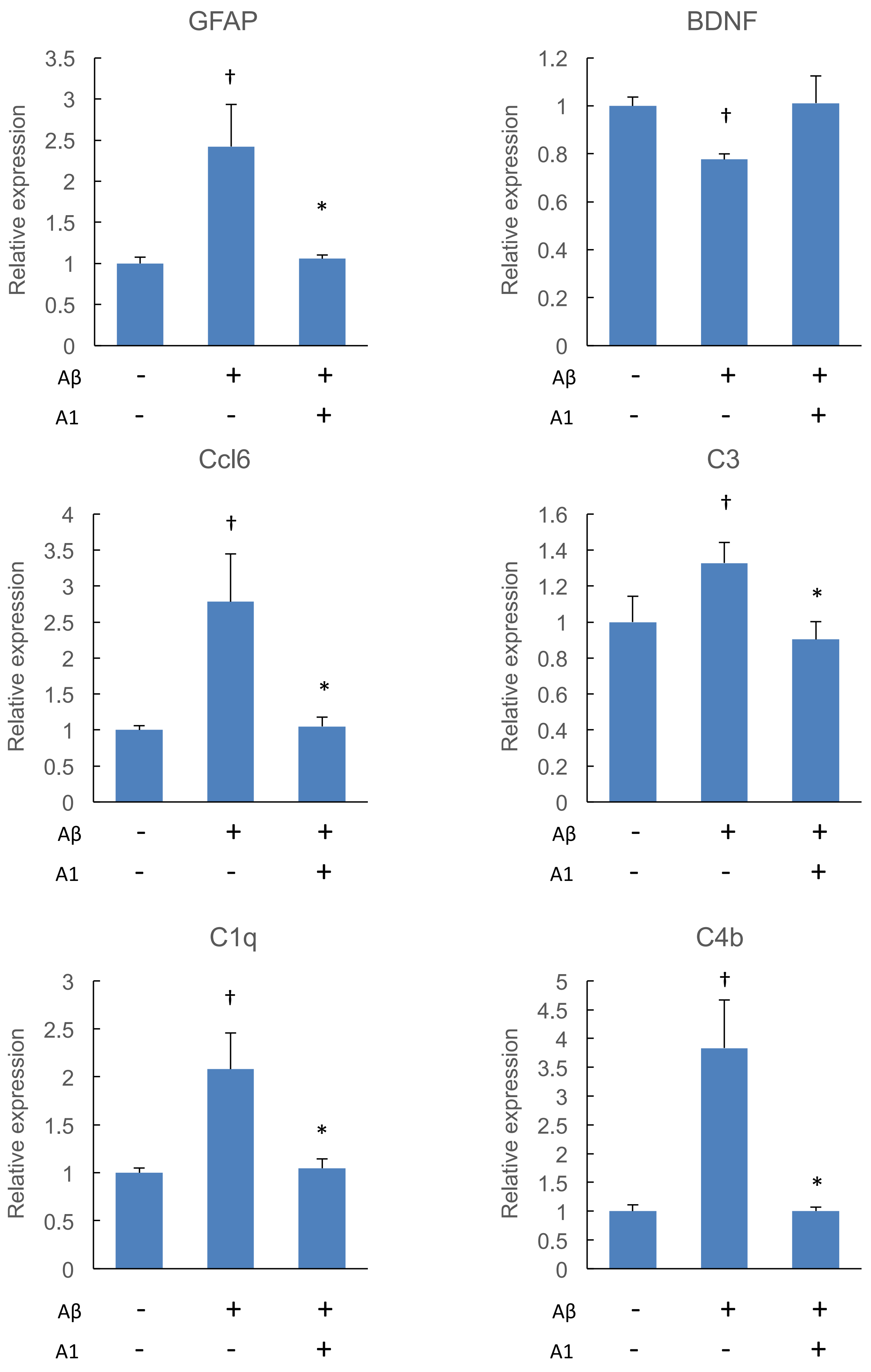

Supplemental Figure 2. Verification of RNA-seq data by quantitative RT-PCR analysis.

mRNA expression of Gfap (A), Ccl6 (B), C1qa (C), C3 (D), C4b (E) and Bdnf (F) in hippocampus. n=5 mice in each group. †$P<0.05$ vs. SH, *$P<0.05$ vs. AB.

Supplemental Fig 3

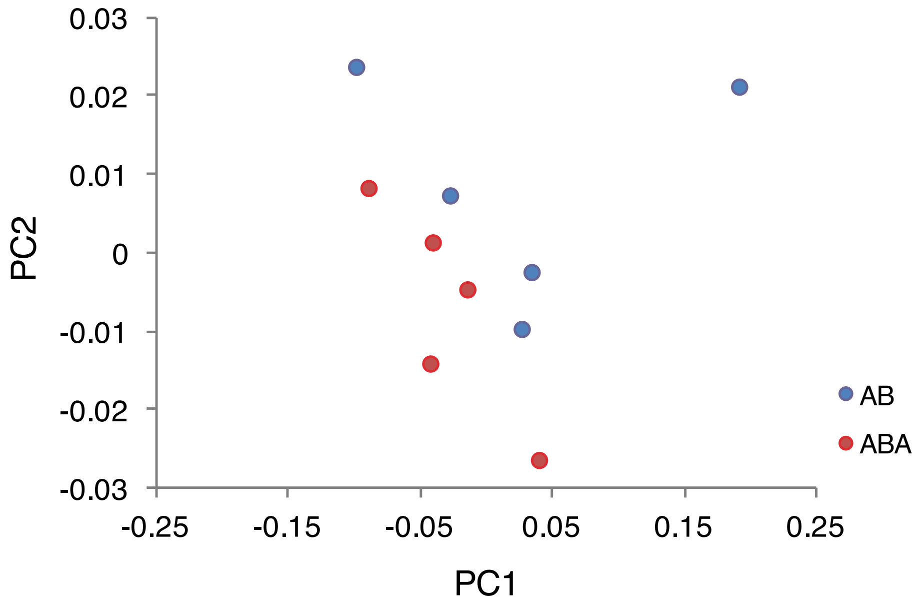

Supplemental Figure 3.

Bray-Curtis dissimilarity based principal coordinates analysis (PCoA) was performed on 16S rRNA genes at phylum level. There was no significant difference between Aβ injected mice (AB) and Aβ injected mice administrated *Bifidobacterium breve* strain A1 (ABA) by using two-way permutation Manova.

Supplemental Fig 4

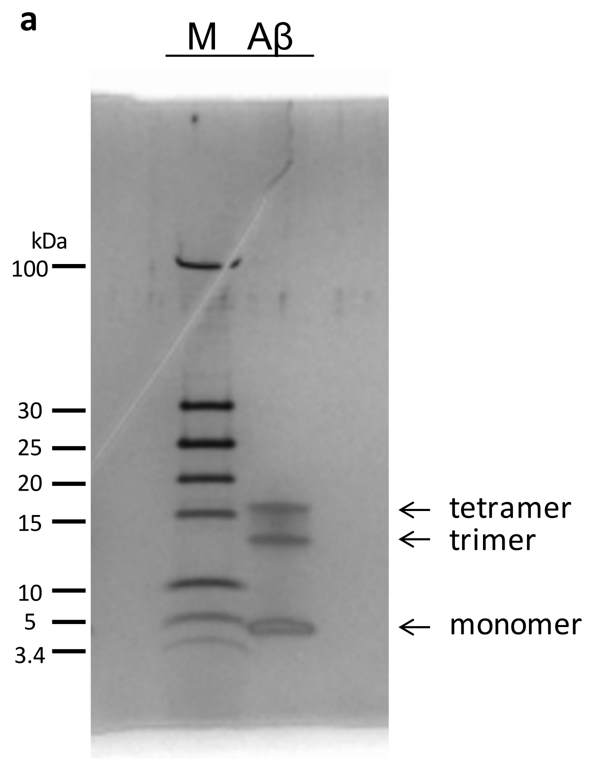

Supplemental Figure 4.

(a) SDS-PAGE and silver-staining are performed to visualize cross-linked Aβ peptide. M: protein ladder marker, Aβ: Aβ1-42 solution. Aβ 1-42 we used in this study produced three bands consistent with monomers, trimers and tetramers. (b) The cropped location in the SDS-PAGE gel was indicated by white dotted line.

Supplemental Table 1: List of gene expression profile in hippocampus of AD model mice by *Bifidobacterium breve* A1 treatment using RNA-seq analysis.

	group	number of genes	gene name
SH vs AB	SH>AB	58	Cntn1, Lrrtm4, Grin2b, Bdnf, Ehd3, Trpc4, Ap2b1, Lrrn1, Taf9_1, D3Bwg0562e, Ate1, Cisd1, Uqcrc2, Ppa1, Usp12, Elmod1, Clvs2, Gcnt2, Dapk1, Pip5k1b, Atp2b3, Cops5, Atp5a1, Wdr61, Gpr22, Ldlr, Dlg3, Hspa4, Ip6k2, Cycs, Emc2, Fdft1, Crbn, A830018L16Rik_1, Gm21955, Paqr9, Dctn3, Nrip3, Atp1b1, Slc16a7, Ap3b1, Fdps, Garem, Cers6, Hmgcr, Rad23b, Grasp, D5Ertd579e, Atp5b, Fam65b, Dnm1l, Fam84a, AC129605.1, Atp6ap2, Mdh1, Ddn, Pls3, Wasf1
	SH<AB	247	Hmha1, Col11a2, Selplg, Slco2b1, Rapgef3, Ccr5, Sspo, Oasl2, Pld4, Glul, Epb4.1l2, Ifit3, Col16a1, Laptm5, Tsc22d4, Sall1, Sepp1, Lag3, C4b, Col24a1, Fes, Gmip, Rnf213, Fcgr1, Csf3r, Trim34a, BC030499, Cx3cr1, Tlr9, Cyth4, Rassf3, Il17ra, Itgam, Cit, Inpp5d, Pik3ap1, Rps6ka1, Trim30a, Unc93b1, Matn4, Scara3, Jak3, Stard9, Parp14, Csf1r, Kcna6, Tlr7, Aifm3, H2-Q7, Slfn8, Irf7, Myo1f, 4632428N05Rik, Slc39a1, Nrbp2, Icam1, Tnfrsf11a, Gpx3, Fyb, Rora, Fgd2, Plcb3, Tcirg1, Slc11a1, Zbp1, Lpcat2, Itpripl2, Dock2, Igtp, Oas1a, Rnf144a, Prdx6, H2-Q2, Sparc, Rasgef1b, Msn, Hepacam, I830012O16Rik, Ifit1, Trim30d, Irf8, Rasal3, Cmklr1, Capg, Nckap1l, Ggta1, Tifab, Gatm, Bhlhe41, Pnpla7, Gbp2b, Vwa1, Lcp1, Gigyf1, Atp1a2, AF251705, Mef2c, Gm15631, Zfp36l1, Igsf9b, Usp18, Siglec1, Adcy7, Lyn, Uba7, Ifitm3, Anxa3, Ptprc, Ly86, Amy1, Grn, Stom, Plekha4, Prex2, Cadm4, Tnfrsf23, Rnase1, C1qc, Appl2, Xaf1, Pros1, Gja1, Slfn9, Vav1, Cst3, Herc6, Gsdmd, Fhod3, Camk1, Il10ra, Sipa1, C1qa, Myo1c, Ifit2, Aldh1l1, Pygm, Rasgef1c, Mpeg1, C1qb, Tgm1, Apoe, Ptpn6, Cyba, Cd84, Itgb2, Mafb, Sparcl1, Flnc, Rims3, Ddx60, Csf2ra, Trim56, Sfxn5, Gnai2, Notch3, Ptafr, Gbp3, Efhd2, Zcchc24, Ifi44, Ccl19, Irf5, Tmem173, Cd14, AI607873, Necap2, Thbs4, H2-Q4, Renbp, Hk3, Asap3, Fcgr3, Zfp36l2, Ly9, Gfap, Srebf1, Rnase4, Hcls1, Wdfy4, Lair1, Tnfrsf1b, Dnm2, Chi3l1, Nfam1, Snhg11, Grrp1, Inppl1, Rarg, Plcd3, Ctss, Gjb6, Ncf4, Rin2, Slc15a3, Plekho1, Arhgap4, Garnl3, Abca9, Cd53, Farp1, 2610507I01Rik, Ifi27l2a, Gm2026, Cited4, Sfi1, H2-Q6, Coro6, Ttyh1, Btk, Flnb, Lilrb4, H2-T10, Cbln2, Cd33, Sgpl1, Clec4a3, Ankrd34c, Mical1, Clu, Slc44a2, Atp1b2, Itpr2, Cyp2j9, Igfbp5, Vim, Cux1, Arhgap30, Slamf8, Sbno2, Gna13, Irf9, Osmr, Psmb8, Ms4a6c, C3ar1, Oplah, Cux2_1, Traf3ip3, Ch25h, Hck, Vasp, Tgfbr2, Il33, R3hdm2, Skap2, Cybb, Itgal
SH vs ABA	SH>ABA	1	Gm6472
	SH<ABA	1	Pole4
AB vs ABA	AB>ABA	196	Laptm5, Slco2b1, Pld4, Cyth4, C4b, Gbp2b, Tsc22d4, Hmha1, H2-Q4, H2-Q7, Ifit3, Oasl2, H2-Q2, Plcb3, Flnc, Lag3, Col11a2, Ly6a, I830012O16Rik, Cx3cr1, Selplg, Rnf213, Csf1r, Ifit2, Slc7a2, Rassf2, Ly86, Ncf4, Ifit1, Col16a1, Themis2, Tmem119, Epb4.1l2, Nckap1l, Zbp1, Slc39a1, Itgam, Cyba, Pla2g7, Pik3ap1, Camk1, Tlr7, 4632428N05Rik, Rab3il1, Gbp9, Psmb8, Uba7, Gsdmd, Wdfy4, Lcp1, Thbd, Tcirg1, Rasal3, Unc93b1, Ikzf1, Gbp3, Myo1f, Fcgr1, Csf3r, Ptplad2, Padi2, Usp18, Irf8, Trim34a, Gfap, Erbb2ip, H2-Q6, Fabp5, Ddx60, Irf7, Xaf1, Stat1, Slc15a3, Cybb, Adhfe1, Dock2, Abhd4, BC035044, St6gal1, Akna, Srebf1, Arpc1b, AI607873, Atp2a3, Eif2ak2, Lpcat2, Msn, Anxa5, C1qa, Capg, Gpam, Zfp217, Tpm4, Ptprc, Gpx3, Tmem173, Dock8, Stard9, Siglec1, Apoe, Cd84, Ednrb, Mlxipl, Tgm1, Iigp1, Ifi44, Gcnt1, Arhgap29, Gbp6, Csf2rb, Slfn8, Parp14, Sall1, Ccl6, Notch3, Cst3, Fes, Fcgr2b, Vat1, Cd300a, Rnf144a, Il10ra, Lyn, Rnase4, Abca9, Hck, Csf1, Icam1, Lrmp, Grn, Fyb, Sfxn5, Gnai2, Ggta1, Ddr1, Asap3, Hepacam, Ptafr, Cd68, Hcls1, Inppl1, Ifitm3, Adcy7, Kcne1l, Thbs4, Vav1, Vim, C3ar1, Mlc1, Gbp7, Trim30d, Slamf8, Phgdh, C1qb, Trim30a, Lilrb4, S1pr3, Myh14, Scara3, Vstm2a, Eml3, Ccr5, Col27a1, Havcr2, Sepp1, Id3, Prex2, Fmnl3, Ctss, Cmklr1, Appl2, Stom, Elf4, Cd86, Igfbp5, Rab32, Clu, Cd52, Prdx6, Clic4, Mpeg1, Gja1, C1qc, Bcl2a1b, Bhlhe41, Tln1, Pnpla7, H2-Q1, Slfn9, Khsrp, Kcna6, Chi3l1, Ly9, Ifi27l2a, Lgals1, Siglech
	AB<ABA	28	Gm5900, Gm6311, Elmod1, Grem1, Arhgap20, Bdnf, 3110035E14Rik, Clvs2, Alox12b, Mal2, N6amt2, Pgm2l1, Atp1b1, Rad23b, Cd47_1, Cntn1, Lca5, Dctn3, AC129605.1, Fhl2, Gabrb2, Pou3f1, BC030500, Cadps2, Tspan13, Ppm1h, Etv1, Fam219a,
	(AB>ABA) ∩ (SH < AB)	130	Laptm5, Slco2b1, Pld4, Cyth4, C4b, Gbp2b, Tsc22d4, Hmha1, H2-Q4, H2-Q7, Ifit3, Oasl2, H2-Q2, Plcb3, Flnc, Lag3, Col11a2, I830012O16Rik, Cx3cr1, Selplg, Rnf213, Csf1r, Ifit2, Ly86, Ncf4, Ifit1, Col16a1, Epb4.1l2, Nckap1l, Zbp1, Slc39a1, Itgam, Cyba, Pik3ap1, Camk1, Tlr7, 4632428N05Rik, Psmb8, Uba7, Gsdmd, Wdfy4, Lcp1, Tcirg1, Rasal3, Unc93b1, Gbp3, Myo1f, Fcgr1, Csf3r, Usp18, Irf8, Trim34a, Gfap, H2-Q6, Ddx60, Irf7, Xaf1, Slc15a3, Cybb, Dock2, Srebf1, AI607873, Lpcat2, Msn, C1qa, Capg, Ptprc, Gpx3, Tmem173, Stard9, Siglec1, Apoe, Cd84, Tgm1, Ifi44, Slfn8, Parp14, Sall1, Notch3, Cst3, Fes, Rnf144a, Il10ra, Lyn, Rnase4, Abca9, Hck, Icam1, Grn, Fyb, Sfxn5, Gnai2, Ggta1, Asap3, Hepacam, Ptafr, Hcls1, Inppl1, Ifitm3, Adcy7, Thbs4, Vav1, Vim, C3ar1, Trim30d, Slamf8, C1qb, Trim30a, Lilrb4, Scara3, Ccr5, Sepp1, Prex2, Ctss, Cmklr1, Appl2, Stom, Igfbp5, Clu, Prdx6, Mpeg1, Gja1, C1qc, Bhlhe41, Pnpla7, Slfn9, Kcna6, Chi3l1, Ly9, Ifi27l2a
	(AB<ABA) ∩ (SH > AB)	8	AC129605.1, Atp1b1, Bdnf, Clvs2, Cntn1, Dctn3, Elmod1, Rad23b

The table presents the list of shared and unique DE genes in SH vs AB, SH vs ABA and/or AB vs ABA. SH: sham-operated mice; AB: Aβ25-35 injected mice; ABA: mice treated with Aβ and *Bifidobacterium breve* A1.

Supplemental Table 2: List of gene expression profile in hippocampus of sham-operated mice by *Bifidobacterium breve* A1 treatment using RNA-seq analysis.

	group	number of genes	gene name
SH vs SHA	SH>SHA	0	–
	SH<SHA	1	Cers1

The table presents the list of shared and unique DE genes in SH vs SHA. SH: sham-operated mice; SHA: sham-operated mice treated with *Bifidobacterium breve* A1.

Supplemental Table 3: PCR primers used in Supplemental Figure 2

	PCR primer
glyceraldehyde-3-phosphate dehydrogenase (Gapdh)	(forward) AAATGGTGAAGGTCGGTGTG
	(reverse) TGAAGGGGTCGTTGATGG
glial fibrillary acidic protein (Gfap)	(forward) GACCAGCTTACGGCCAACAG
	(reverse) TCTATACGCAGCCAGGTTGTTCTC
chemokine (C-C motif) ligand 6 (Ccl6)	(forward) AGAAACTCCAAGACTGCCATTTCA
	(reverse) TTTACATGGGATCTGTGTGGCATA
complement component 1, q subcomponent, alpha polypeptide (C1qa)	(forward) CATCCAGTTTGATCGGACCAC
	(reverse)AGACATCTTCAGCCACTGTCCATAC
complement component 3 (C3)	(forward) CCTGTAGTGCGCTGGCTCAA
	(reverse) CAAGTCCTTATGGTCAGGGACATC
complement component 4B (C4b)	(forward) ACCGTTACGTGAGTCACTTTGAGA
	(reverse) ATACAGGACAGCACTGGATGG
brain derived neurotrophic factor (Bdnf)	(forward) TCAAGTTGGAAGCCTGAATGAATG
	(reverse) CTGATGCTCAGGAACCCAGGA
